# Supplementary material for: Core species and interactions prominent in fish-associated microbiome dynamics
Source: Microbiome. 2023 Mar 20;11:53. doi: 10.1186/s40168-023-01498-x (PMC10026521; doi:10.1186/s40168-023-01498-x)
Supplement: Supplementary file 3 — Additional file 2: Figure S2. Order-level community structure. [file 40168_2023_1498_MOESM2_ESM.docx]

**Additional file 2: Fig. S2** Order-level community structure. **a** Dynamics of absolute abundance. For each water sample of each aquaculture tank, absolute abundance of prokaryotes was inferred as 16S rRNA gene copy concentration based on the quantitative amplicon sequencing approach with standard DNA gradients. **b** Dynamics of relative abundance. The time-series of the order-level taxonomic compositions are shown for each aquaculture tank.

**
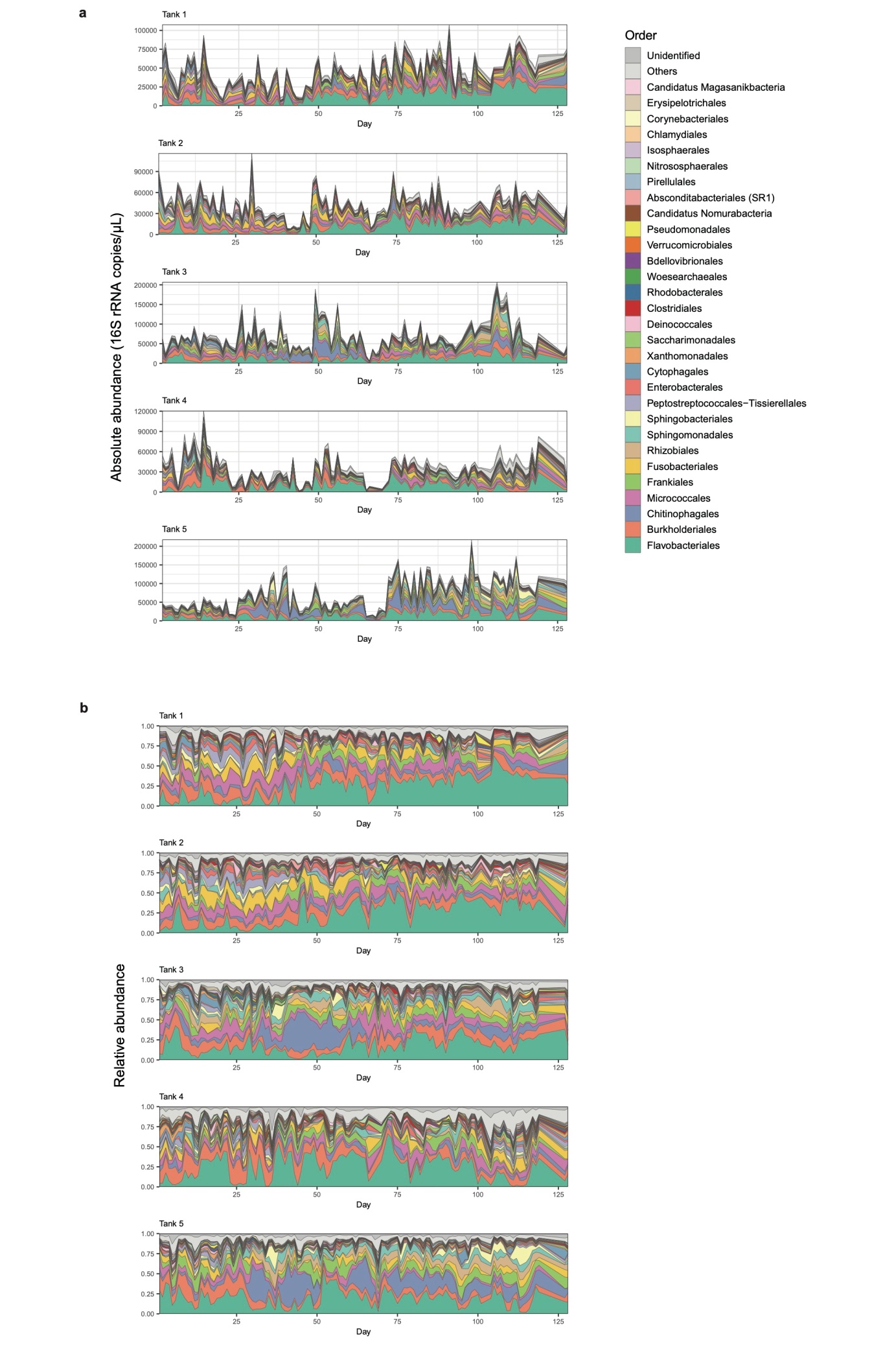
**
