# Supplementary material for: Core species and interactions prominent in fish-associated microbiome dynamics
Source: Microbiome. 2023 Mar 20;11:53. doi: 10.1186/s40168-023-01498-x (PMC10026521; doi:10.1186/s40168-023-01498-x)
Supplement: Supplementary file 5 — Additional file 4: Figure S4. Multivariate analysis of community structure (genus level). [file 40168_2023_1498_MOESM4_ESM.docx]

**Additional file 4: Fig. S4** Multivariate analysis of community structure (genus level). **a** Community state space. Community compositions of the samples are plotted on the two-dimensional surface defined with non-metric multidimensional scaling (NMDS). The NMDS was performed based on the Bray-Curtis *β*-diversity of genus-level taxonomic compositions. The projections of the data points onto the vectors have maximum correlation with the variables examined (pH, DO, and eels’ activity level). **b** Examples of community structure in the NMDS surface. For several points within the NMDS surface (panel **a)**, genus-level taxonomic compositions are shown. The example points are ordered along the vector representing high eels’ activity level. **c** Indicator genera. The vectors representing the relative abundance of *Cetobacterium*, *Flavobacterium*, and *Edaphobaculum*, which were highlighted in the main text, are shown.

**
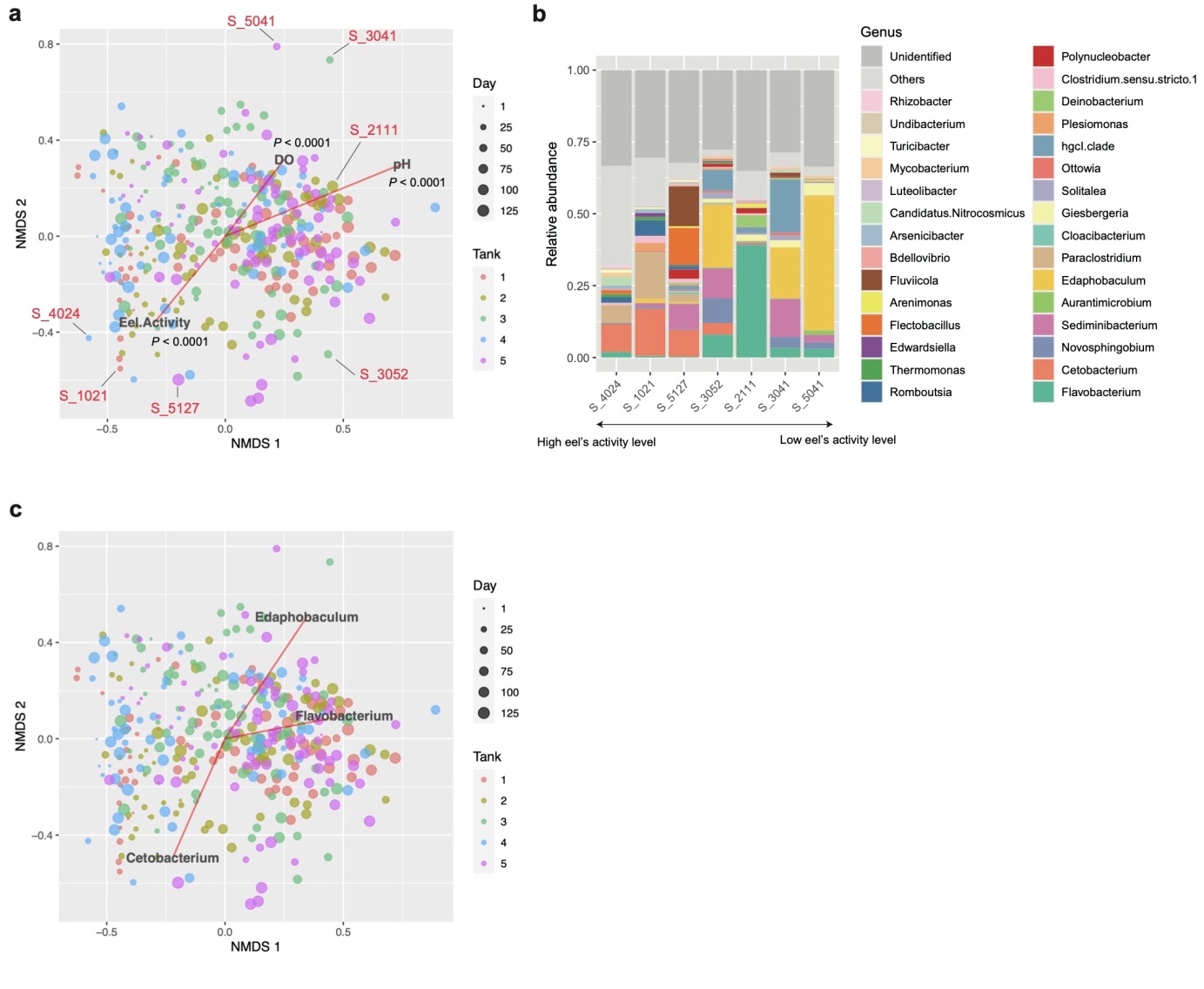
**
