# Supplementary material for: Core species and interactions prominent in fish-associated microbiome dynamics
Source: Microbiome. 2023 Mar 20;11:53. doi: 10.1186/s40168-023-01498-x (PMC10026521; doi:10.1186/s40168-023-01498-x)
Supplement: Supplementary file 6 — Additional file 5: Figure S5. ASV-level comparison of correlation with environmental variables and eels’ activity level. [file 40168_2023_1498_MOESM5_ESM.docx]

**Additional file 5: Fig. S5** ASV-level comparison of correlation with environmental variables and eels’ activity level. **a** Correlation with pH. Correlation with eels’ activity level is shown for the ASVs that appeared in all the aquaculture tanks (shown in the decreasing order of mean values). The boxes and bars represent variation across tanks. **b** Correlation with DO. **c** Correlation with eels’ activity level. **d** Partial correlation with eels’ activity level (controlled variable = pH). Taxonomic information is shown for the ASVs discussed in the main text.

**
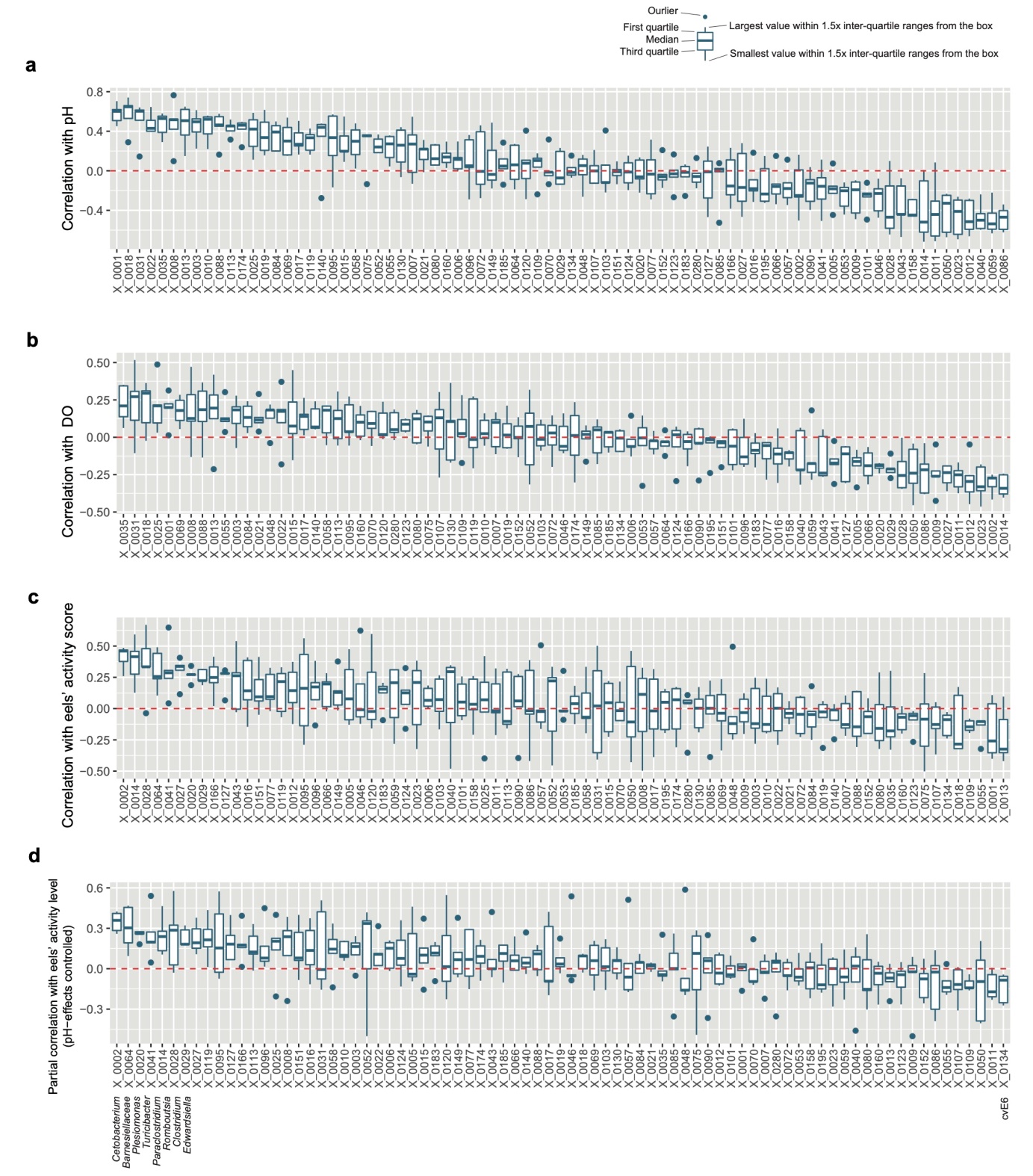
**
