# Supplementary material for: Core species and interactions prominent in fish-associated microbiome dynamics
Source: Microbiome. 2023 Mar 20;11:53. doi: 10.1186/s40168-023-01498-x (PMC10026521; doi:10.1186/s40168-023-01498-x)
Supplement: Supplementary file 7 — Additional file 6: Figure S6. Phylogenetic analysis of Edwardsiella. [file 40168_2023_1498_MOESM6_ESM.docx]

**Additional file 6: Fig. S6** Phylogenetic analysis of *Edwardsiella*. **a** Phylogeny of *Edwardsiella.* In an additional amplicon sequencing of the *sodB* gene, the neighbor-joining tree of the *Edwardsiella* bacteria was reconstructed with the maximum composite likelihood method. Bootstrap values larger than 70 % are shown on the nodes (1,000 permutations). The pathogenic and non-pathogenic clades identified in a previous study [28] are indicated. **b** Time-series of pathogenic and non-pathogenic *Edwardsiella.* The number of detected sequencing reads of the *sodB* fragment is across the time-series of each aquaculture tank.

**
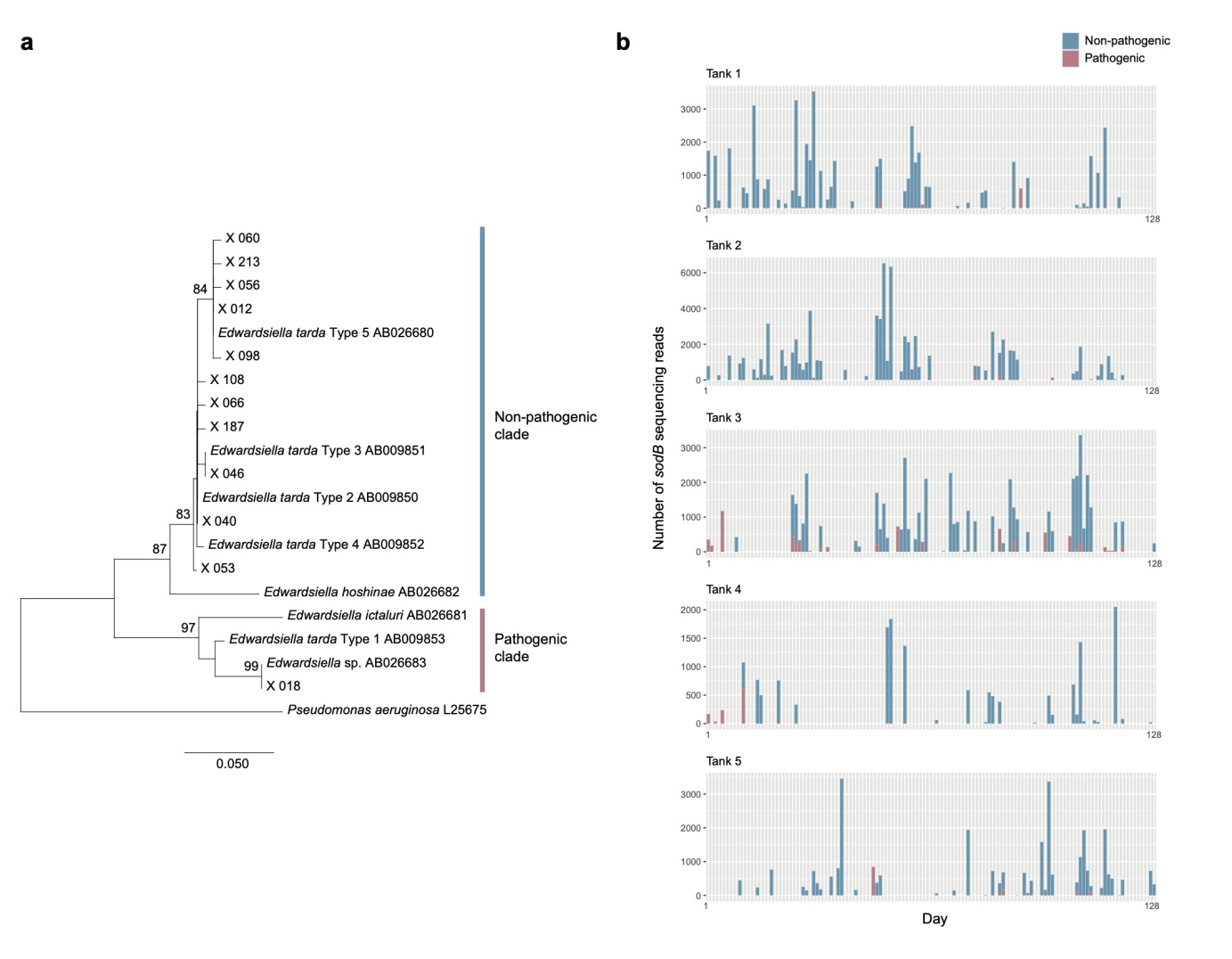
**
