# Supplementary material for: Core species and interactions prominent in fish-associated microbiome dynamics
Source: Microbiome. 2023 Mar 20;11:53. doi: 10.1186/s40168-023-01498-x (PMC10026521; doi:10.1186/s40168-023-01498-x)

**Additional file 7: Fig. S7** Taxonomy of the nodes within the coexistence networks. Within the coexistence networks shown in Figure 4, phylum-level taxonomy of the ASVs is shown. ASVs included in minor sub-networks (number of nodes < 5) are not shown. Only the ASVs that appeared in 30 or more samples were targeted in the analysis of each tank.


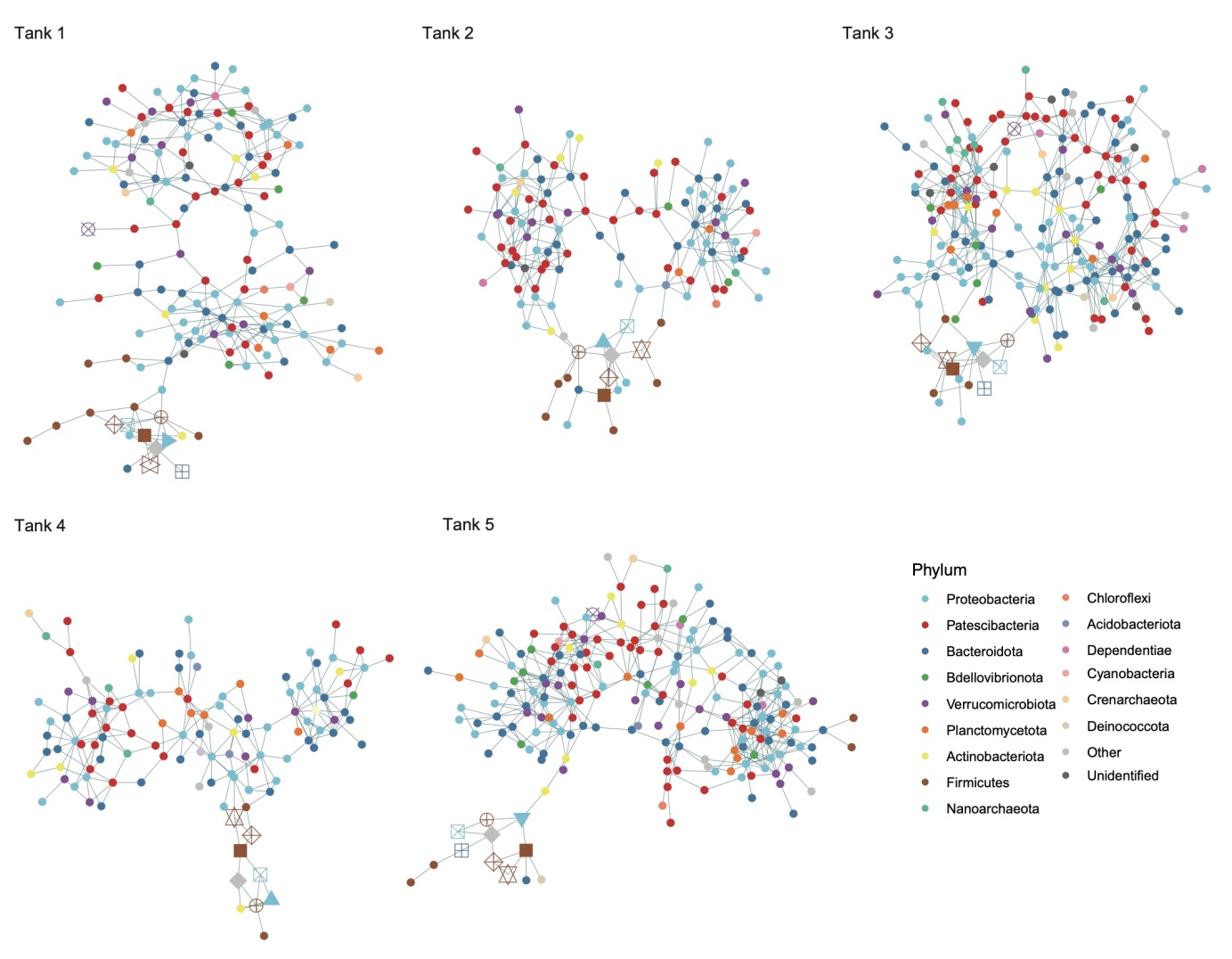

Supplement: Supplementary file 8 — Additional file 7: Figure S7. Taxonomy of the nodes within the coexistence networks. [file 40168_2023_1498_MOESM7_ESM.docx]
