# Supplementary material for: Core species and interactions prominent in fish-associated microbiome dynamics
Source: Microbiome. 2023 Mar 20;11:53. doi: 10.1186/s40168-023-01498-x (PMC10026521; doi:10.1186/s40168-023-01498-x)
Supplement: Supplementary file 9 — Additional file 8: Figure S8. Correlations with environmental variables. [file 40168_2023_1498_MOESM8_ESM.docx]

**Additional file 8: Fig. S8** Correlations with environmental variables. **a**, Correlation with pH. For each microbial ASV included within the coexistence network of each aquaculture tank (Fig. 4), correlation between absolute abundance and pH is shown. ASVs included in minor sub-networks (number of nodes < 5) are not shown. Only the ASVs that appeared in 30 or more samples were targeted in the analysis of each tank. **b**, Correlation with dissolved oxygen level. For each microbial ASV included within the coexistence network of each aquaculture tank (Fig. 4), correlation between absolute abundance and dissolved oxygen (DO) level is shown.

**
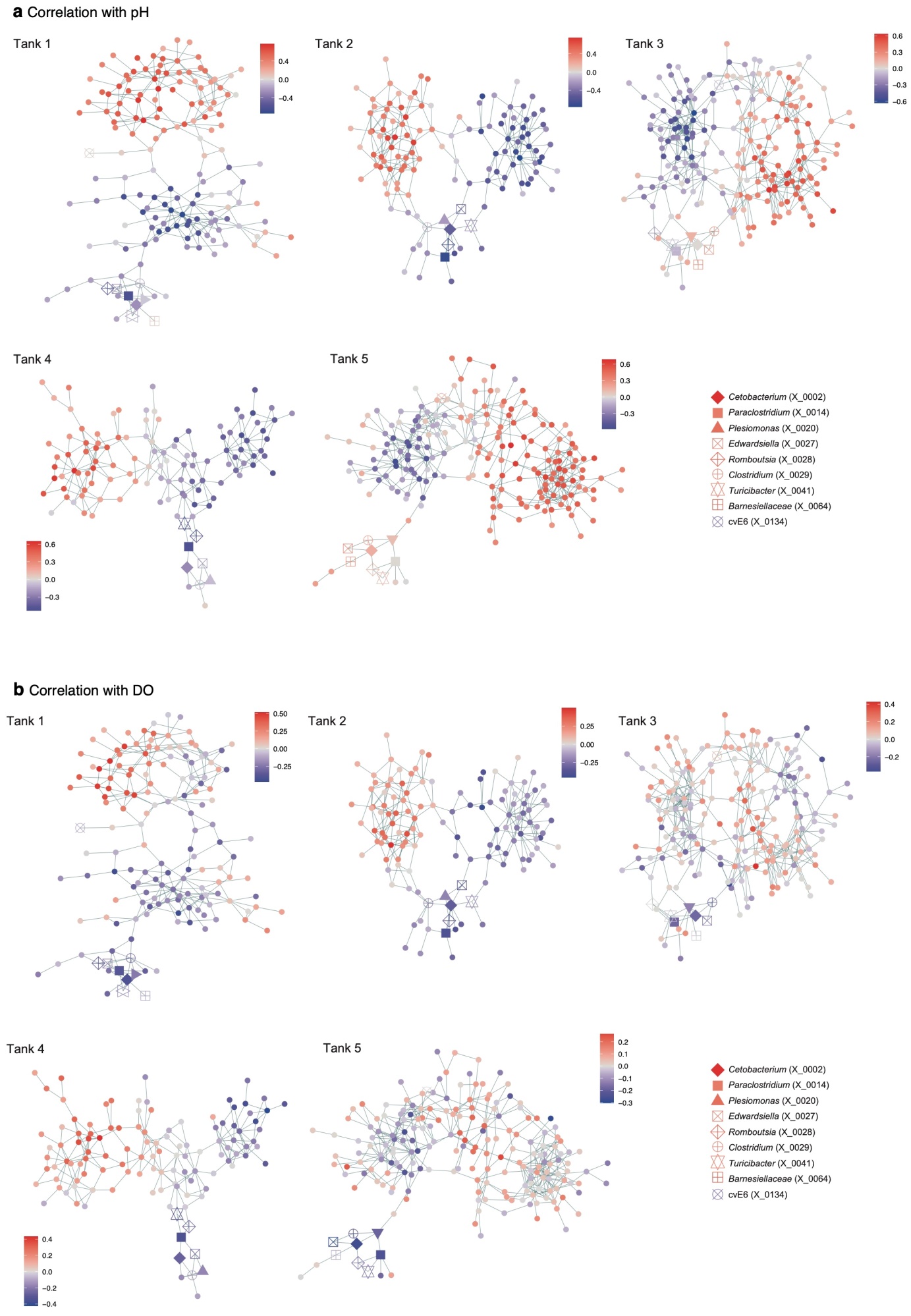
**
