# Supplementary material for: Core species and interactions prominent in fish-associated microbiome dynamics
Source: Microbiome. 2023 Mar 20;11:53. doi: 10.1186/s40168-023-01498-x (PMC10026521; doi:10.1186/s40168-023-01498-x)

**Additional file 10: Fig. S9** Properties of network modules. **a** Module size and mean partial correlation with eels’ activity level. For each module within the coexistence network of each aquaculture tank (Fig. 4), the number of ASVs and mean partial correlation with eels’ activity level are shown. The modules including the *Cetobacterium* ASV (X_0002) is indicated by arrows. The outlier modules with large numbers of constituent ASVs and low/high mean partial correlation with eels’ activity level are highlighted by circles. **b** Modules including the *Cetobacterium* ASV (X_0002). The top-five ASVs with the highest partial correlation with eels’ activity level are shown for each module. **c** Outlier modules with high mean partial correlation with eels’ activity level. **d** Outlier module with low mean partial correlation with eels’ activity level.


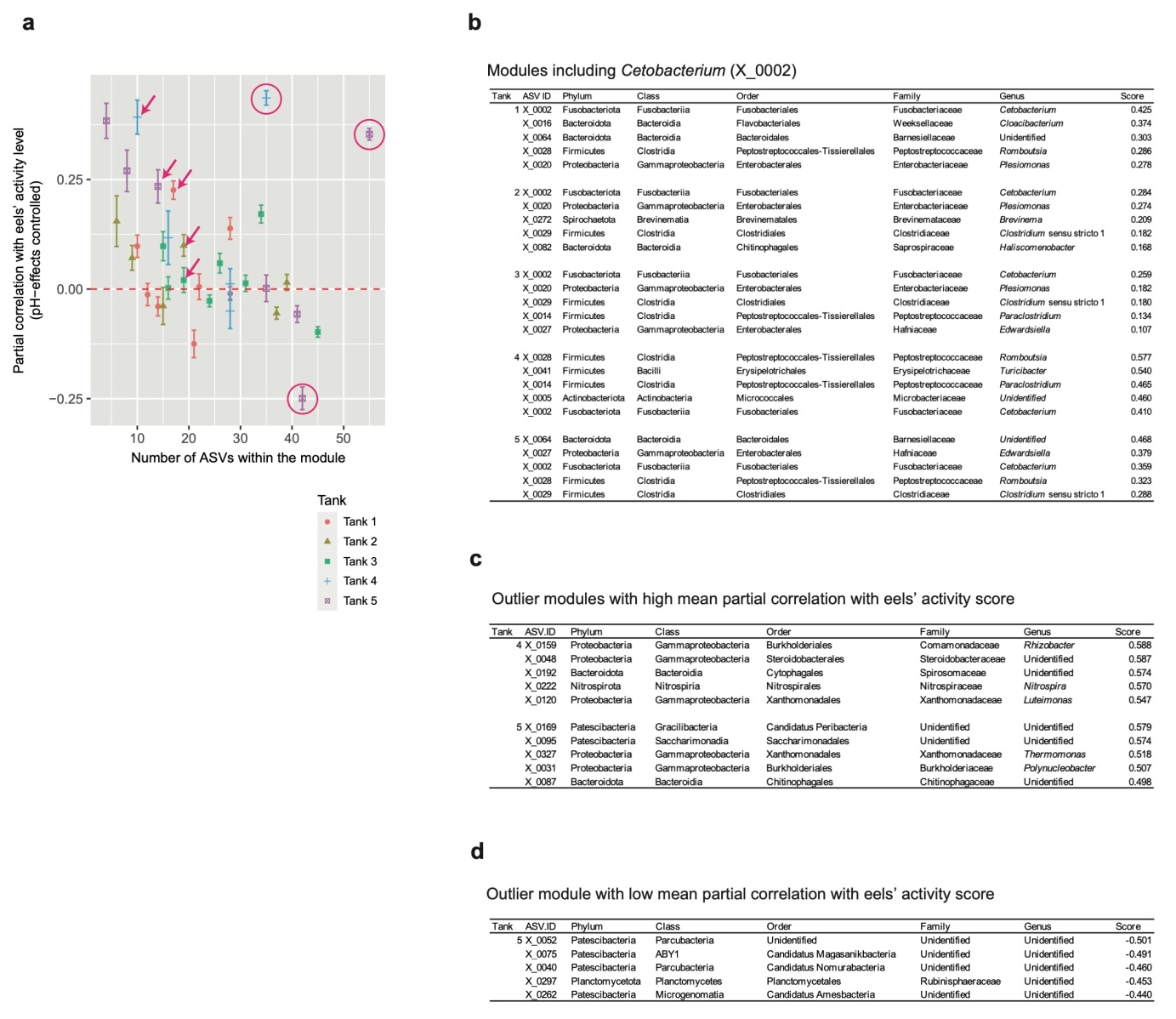

Supplement: Supplementary file 11 — Additional file 10: Figure S9. Properties of network modules. [file 40168_2023_1498_MOESM10_ESM.docx]
