# Supplementary material for: Core species and interactions prominent in fish-associated microbiome dynamics
Source: Microbiome. 2023 Mar 20;11:53. doi: 10.1186/s40168-023-01498-x (PMC10026521; doi:10.1186/s40168-023-01498-x)

**Additional file 11: Fig. S10** Comparison of network reconstruction methods. For each aquaculture tank, the network links inferred with the MB method was compared with those inferred with the SLR methods. The former is expected to represent interspecific interactions as well as potential sharing of environmental preference (i.e., niches) between nodes (ASVs). Meanwhile, the latter is expected to represent direct interactions between nodes. A positive/negative value indicates a potentially positive/negative interaction between a pair of microbial ASVs. The positive values (> 0) were used to draw networks of potential positive interactions between microbes as shown in Figure 5.


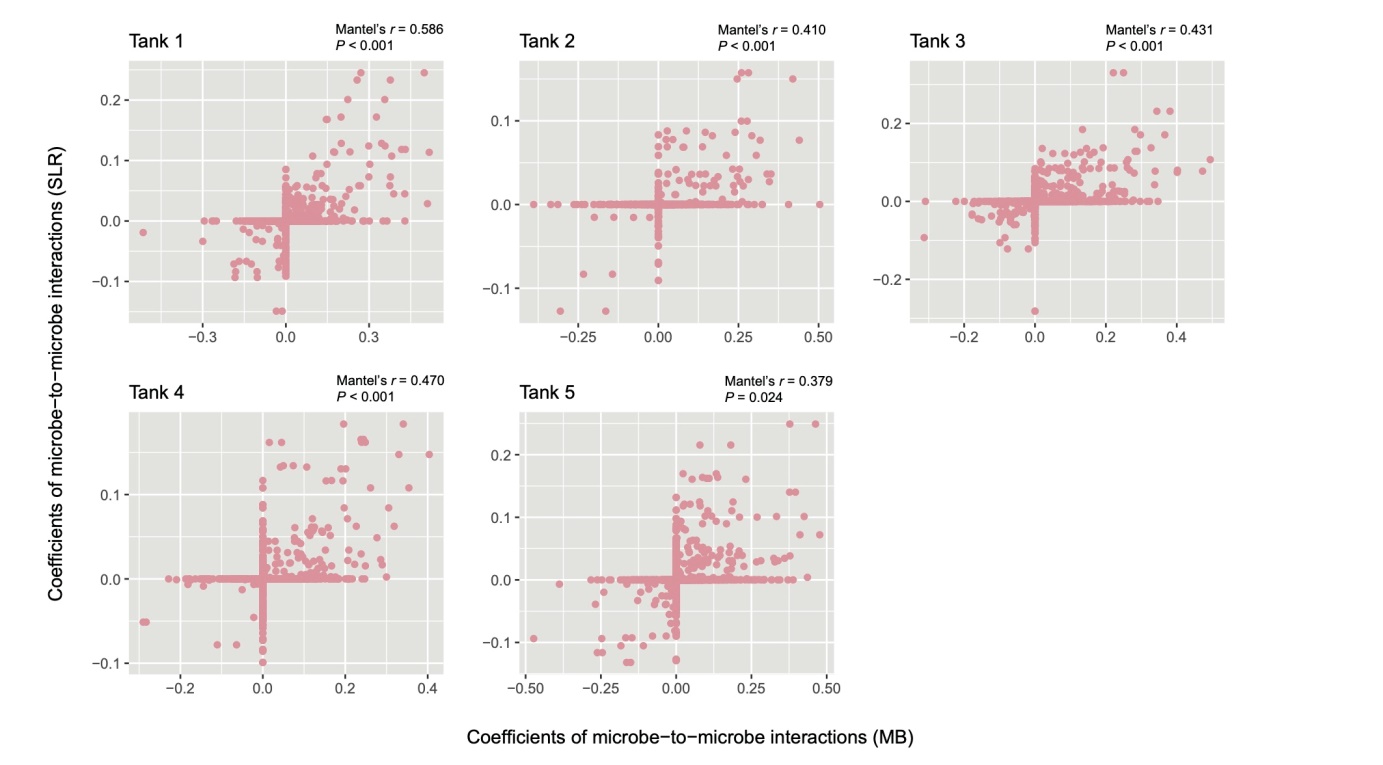

Supplement: Supplementary file 12 — Additional file 11: Figure S10. Comparison of network reconstruction methods. [file 40168_2023_1498_MOESM11_ESM.docx]
