# Supplementary material for: Core species and interactions prominent in fish-associated microbiome dynamics
Source: Microbiome. 2023 Mar 20;11:53. doi: 10.1186/s40168-023-01498-x (PMC10026521; doi:10.1186/s40168-023-01498-x)

**Additional file 12: Fig. S11** Rarefaction curves of the samples. For each sample, relationship between the number of sequencing reads and the number of amplicon sequence variants is shown.


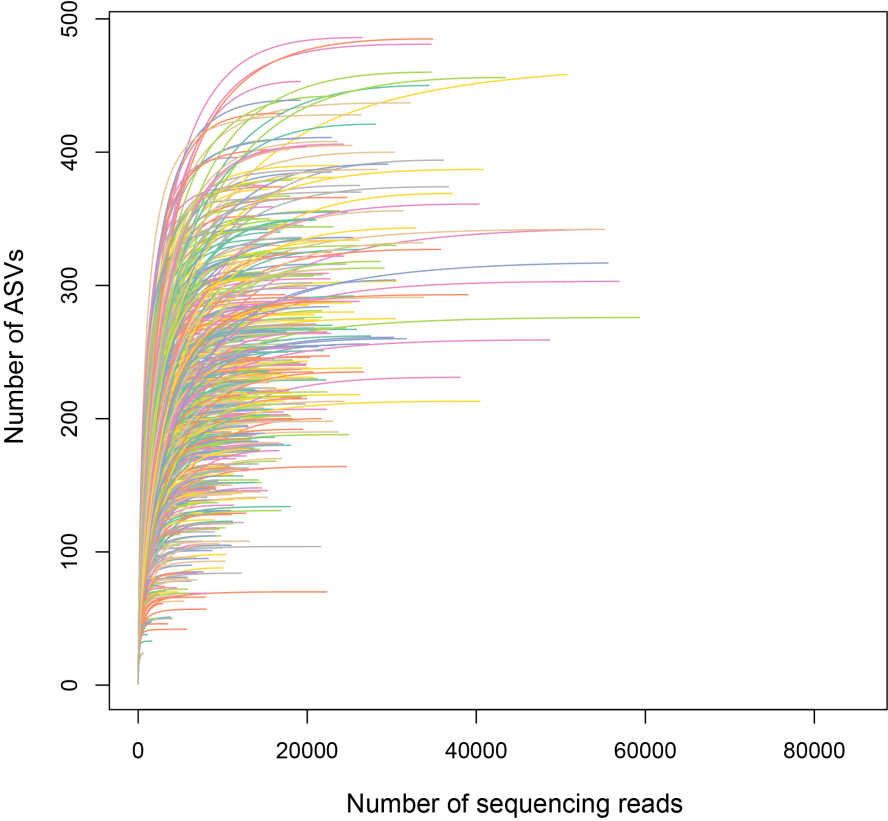

Supplement: Supplementary file 13 — Additional file 12: Figure S11. Rarefaction curves of the samples. [file 40168_2023_1498_MOESM12_ESM.docx]
