# Supplementary material for: Core species and interactions prominent in fish-associated microbiome dynamics
Source: Microbiome. 2023 Mar 20;11:53. doi: 10.1186/s40168-023-01498-x (PMC10026521; doi:10.1186/s40168-023-01498-x)

**Additional file 13: Fig. S12** Temporal dynamics of potential fish pathogens. For each aquaculture tank, the total absolute abundance (16S rRNA gene copy concentrations) of bacterial ASVs belonging to the genera including notorious fish pathogens is shown through the time-series.


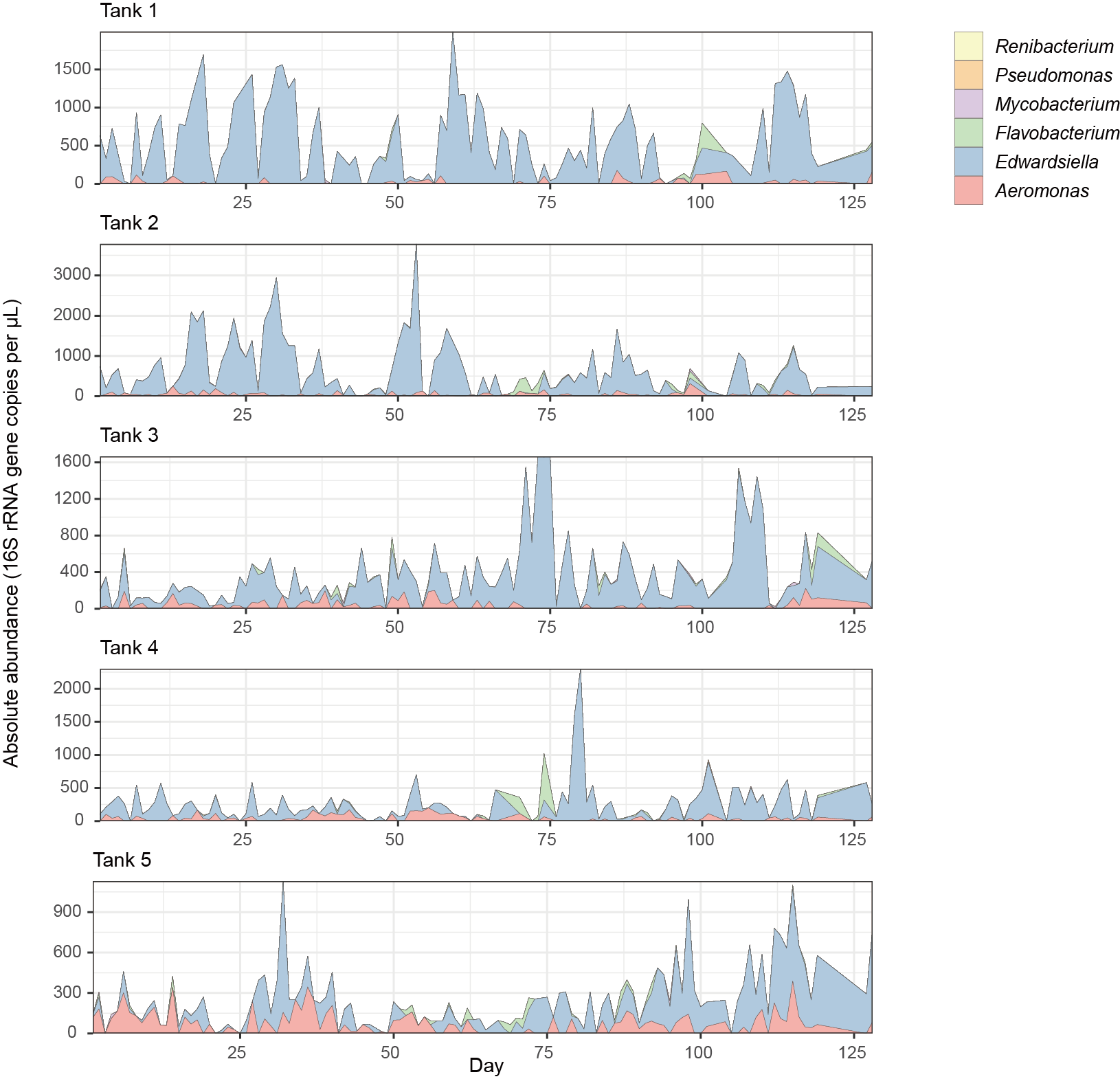

Supplement: Supplementary file 14 — Additional file 13: Figure S12. Temporal dynamics of potential fish pathogens. [file 40168_2023_1498_MOESM13_ESM.docx]
